# Generation of all-male-like sterile zebrafish by eliminating primordial germ cells at early development

**DOI:** 10.1038/s41598-018-20039-3

**Published:** 2018-01-30

**Authors:** Li Zhou, Yongyong Feng, Fang Wang, Xiaohua Dong, Lan Jiang, Chun Liu, Qinshun Zhao, Kaibin Li

**Affiliations:** 1 Pearl River Fishery Research Institute, Chinese Academy of Fishery Sciences, No. 1 Xingyu Road, Xilang, Liwan District, Guangzhou, Guangdong, 510380 China; 2 MOE Key Laboratory of Model Animal for Disease Study, Model Animal Research Center, Nanjing University, 12 Xuefu Road, Pukou High-tech Development Zone, Nanjing, Jiangsu 210061 China

## Abstract

Production of all-male and sterile fish may not only substantially improve yield but also be crucial for the application of genome modified species in aquaculture. Previously, it was reported that the fish lacking primordial germ cells (PGCs) becomes infertile, and nitroreductase, an enzyme converting non-toxic metronidazole (MTZ) into toxic metabolites, induces targeted toxicity to kill the cells expressing it. In this study, we generated a transgenic zebrafish line of Tg(*nanos3*:nfsB-mCherry-*nanos3* 3′UTR) in which the NfsB nitroreductase is solely expressed in PGCs. Treating the embryos derived from the female transgenic zebrafish with MTZ from 0 through 2 dpf (days post fertilization), we found that the germ cells were completely eliminated in the ones older than 2.5 dpf. At 20 dpf, the MTZ-treated juvenile had no germ cells in their gonads. At 100 dpf, the MTZ-treated adult exhibited male-like morphology and showed normal mating behaviors although they had no germ cells but only supporting cells in their gonads. Taken together, our results demonstrated that conditional elimination of PGCs during early development make the zebrafish male-like and infertile. It may provide an alternative strategy to make sterile and all-male farmed fish that is good for increasing aquaculture yield and preventing the genome modified species from potential ecological risks.

## Introduction

Infertility treatment of farmed fish is a promising strategy for increasing aquaculture production as well as mitigating the potential ecological risks from biological invasion by escaped farmed species^1^. In addition, inhibiting the development of sex organs can improve fish growth, enhance the quality of aquaculture products, increase the utilization rate of feed, and reduce production costs^2^. Species-specific infertile techniques are therefore desirable for efficient large-scale farming^1^.

Fish are sterilized by eliminating germ cells or interfering with reproductive function. The polyploid induction technique is frequently utilized in aquaculture, especially for rainbow trout (*Oncorhynchus mykiss*)^3^ and crucian carp (*Carassius carassius*)^4^. The sterile individuals obtained by this technique are the offspring of multiple parental hybridizations with different ploidies. The germ cells may become infertile due to chromosome synapsis disorder during meiosis^5^, which can be maintained by “fertile parent and infertile filial generation”^1^. Surgery, drug treatment, and morpholino targeting reproductive cells have also been utilized to make fish infertile^2^. However, these strategies have poor specificity, low efficiency, and are thus unsuitable for large-scale aquaculture production.

The germ cells of fish are derived from primordial germ cells (PGCs), so loss of PGCs impedes gonadal development^6^. Transgenic techniques causing stagnation of PGC development offer an alternative sterilization strategy. The Maternal Sterility Technology (MST) employs maternal expression of a pro-apoptotic protein to eliminate PGCs and obtain infertile individuals^7^, but the efficacy of this pro-apoptotic protein on early development has not been assessed in practice. It is also challenging to maintain infertile parant^8^. Disrupting PGC migration during the early stage of development is another promising infertility technique for industrialized application. For instance, Wong *et al*. overexpressed stromal cell-derived factor 1a (Sdf1a), a chemokine vital for PGC homing, in zebrafish by heat induction, resulting in mis-migration of the PGCs and the development of sterile fish^9^.

Nitroreductase (NTR), an enzyme derived from *Escherichia coli*, can convert non-toxic metronidazole (MTZ) into toxic metabolites, thereby inducing targeted toxicity in fish cells expressing NTR^10^. Indeed, this NTR/MTZ system is frequently employed for targeted ablation in studies of organ regeneration and function. For instance, it has been applied to conditionally eliminate ventricular cardiomyocytes^11^, hepatocytes^12^, and pancreatic β cells^13^ in zebrafish (*Danio rerio*) to investigate the mechanism underlying organ repair following injury. In addition, it can be applied for the targeted elimination of gonadal cells. Hsu *et al*. utilized the promoters of *asp*, *odf*, and *sam* to specifically drive the expression of the NTR coding gene *nfsB* in zebrafish gonadal tissues and found that MTZ treatment could induce male infertility^14^. Dai *et al*. employed *dnd* promoter to construct NTR transgenic fish and demonstrated that MTZ treatment could induce male transformation and severely impair reproduction capability^15^.

A series of marker genes, including the maternal genes *vasa*^16^, *piwi*^17^, and *dazl*^18^, have been identified that allow for study of fish PGC origin, migration, and differentiation. The maternal gene *nanos3* encoding the RNA-binding protein is also specifically expressed in PGCs. In this study, we used the zebrafish *nanos3* regulatory sequences driving the expression of NfsB-mCherry specifically in PGCs and examined the effect NTR/MTZ system on zebrafish gonad development. Our results showed that conditionally eliminating PGCs during early development completely ablate the development of germ cells in gonad, resulting in sterile and male-like adult zebrafish.

## Results

### Generation of Tg(*nanos3*:nfsB-mCherry-nanos3 3′UTR) zebrafish

**Figure 1:**
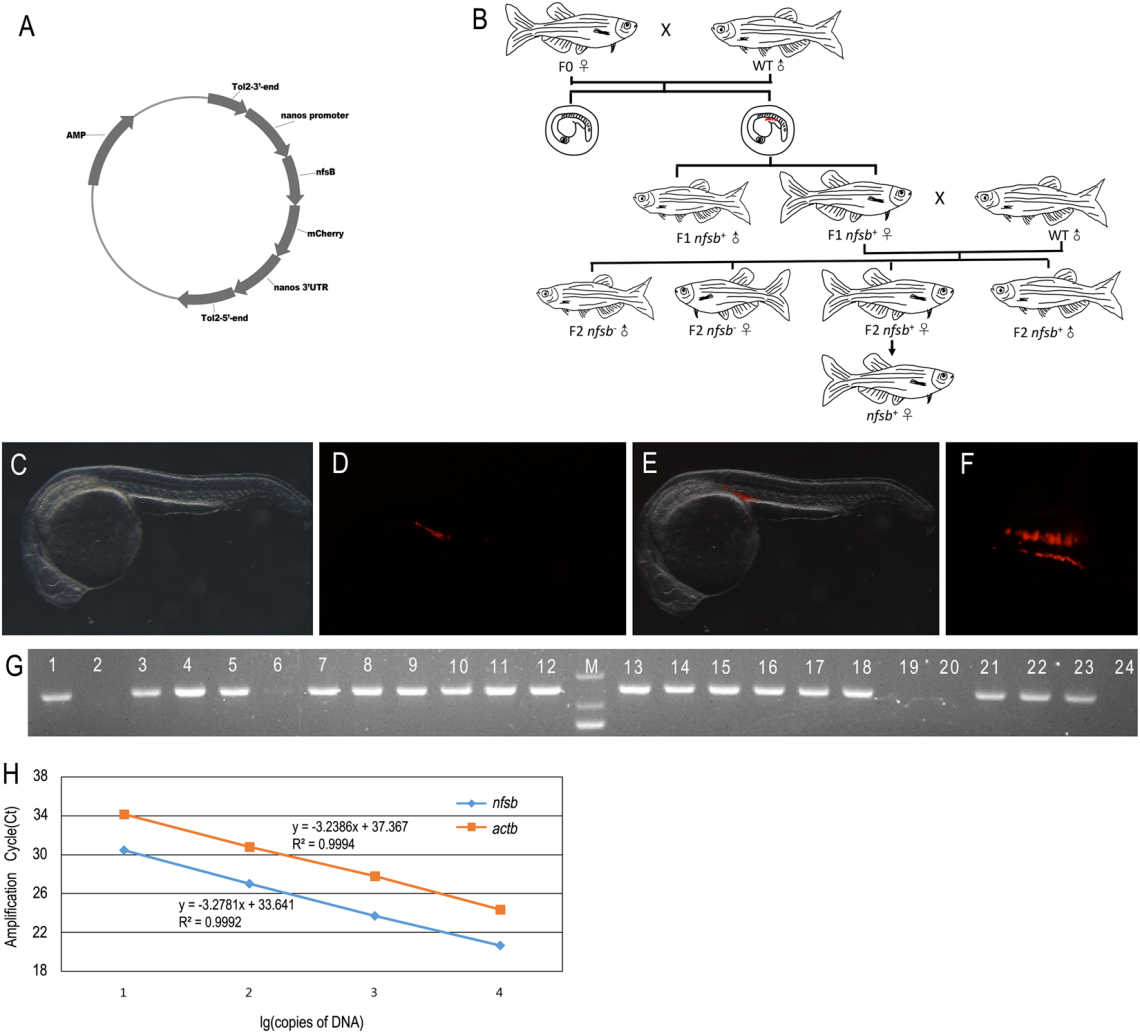
Generation of transgenic zebrafish of Tg(*nanos3*:nfsB-mCherry-nanos3 3′UTR). (**A**) Schematic diagram showing the construct used to generate the transgenic zebrafish line of Tg(*nanos*3:nfsB-mCherry-nanos3 3′UTR). (**B**) Schematic diagram showing the work flow of setting up the transgenic zebrafish. (**C**) Genotyping results of F1 zebrafish by PCR amplification. The gel picture was cropped from an original full-length gel image provided in Supplementary information. The identities of PCR products were further confirmed by Sanger sequencing. (**D**–**F**) Photomicrographs of the typical F2 embryos at 24 hpf displaying the expression of mCherry at the genital ridge (D: light; E: fluorescence; F: merge). Embryos were observed laterally. (**G**) PCGs with fluorescence were arranged as two lines. Embryos were observed dorsally. (**H**) Standard curve comparison. Standard curves for transgene Tg(*nano3*:nsfb-mCherry nanos3 3′UTR) and the endogenous β-actin gene (*actb*) in serially diluted (10-fold) DNA samples from the cloning vector of Tg(*nano3*:nsfb-mCherry nanos3 3′UTR) and wild type zebrafish genome. A very efficient amplification was obtained, as indicated by the slopes of the standards curves. Ct, cycle threshold.

To generate the transgenic zebrafish of Tg(Tol2-*nanos3*-nfsB-mCherry-nanos3 3′UTR), we microinjected the transgenic construct (Fig. 1A) together with transponase mRNA into zebrafish fertilized eggs. Totally, we obtained 80 F0 zebrafish. To obtain the germline transmission transgenic zebrafish, we screened the F1 embryos using the strategy described in Fig. 1B. Briefly, 20 sexual mature females of the F0 were mated with wild-type males to produce F1 embryos. When reaching 24 hpf, the F1 embryos were examined whether they had the mCherry expression under fluorescent microscopy. Among the founders, 18 did not produce any fluorescent embryos. Only Founder #9 and Founder #13 produced 54 of 228 and 24 of 159 embryos carrying the fluorescent signals respectively. Among the embryos, the ones derived from Founder #9 had stronger fluorescent intensity than the ones from Founder #13. We therefore raised the fluorescent F1 embryos produced from Founder #9 for setting up the germline transmission transgenic zebrafish.

At 2 months after fertilization, the caudal fins of F1 zebrafish derived from Founder #9 were clipped to prepare the genomic DNA template for genotyping with PCR amplification using *nfsb* gene-targeted specific primers. Results from agarose gel electrophoresis of the PCR products revealed that 19 of 24 F1 zebrafish were positive for the expected 602 bp product (Fig. 1C) and the transgene identities were further confirmed by Sanger sequencing. The transgenic zebrafish were named Tg(*nanos3*:nfsB-mCherry-nanos3 3′UTR) or *prfri001*. That the other 5 F1 did not carry the transgene but exibit fluorescent at early developemnt is due to the expression of marternal *nsfB-mCherry* transcript.

To examine the expression of the transgene, the female F1 carrying transgene *nfsB-mCherry* was selected to mate with male wild type for producing F2 embryos. As shown in Fig. 1D–F, the localized mCherry fluorescence was found in the genital ridge in the F2 embryos at 24 hpf. Fluorescent cells were arranged and aggregated in two lines (Fig. 1G).

We next performed quantitative PCR to determine how many copies of transgene were recombined into the transgenic zebrafish genome using the method as described previously^19,20^. Briefly, we first set up the standard curve comparison between the copies of housing keeping *actb* DNA and the transgene Tg(Tol2-*nanos3*-nfsB-mCherry-nanos3 3′UTR) verse their PCR cycles (Fig. 1H). We then performed the quantitative PCR using the template isolated from the F2 zebrafish carrying the transgene. The experiments were repeated three times and resulted in an average Ct with 21.773 for the transgenic *nsfb*. Calculated with the standard curve, we found the copy number of the transgenic *nsfb* is about 0.55. Therefore, we concluded that only one copy of transgene were inserted into the genome of transgenic zebrafish *prfri001*.

### The transgenic zebrafish embryos treated with MTZ exhibit ablation of PGCs

**Figure 2:**
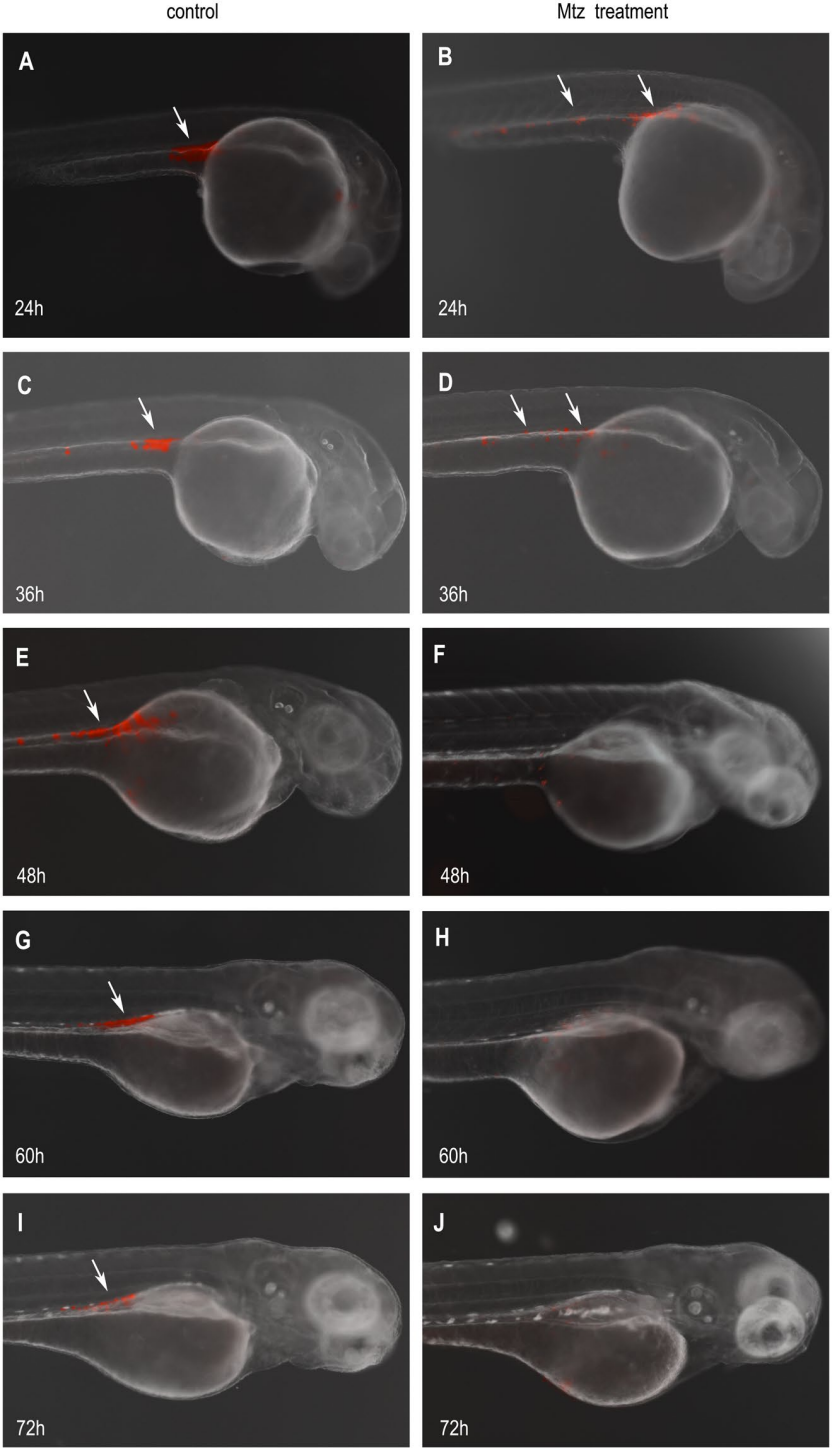
MTZ treatment eliminating the PGCs in the transgenic embryos. The PGCs (white arrow) marked with mCherry fluorescent were around anterior part of the yolk extension in Tg(*nanos3*:nfsB-mCherry-nanos3 3′UTR) transgenic embryos at 24 hpf (**A**), and were almost arranged in the genital ridge at 36, 48, 60, and 72 hpf (**C**,**E**,**G**,**I**), respectively. After treated with MTZ, the 24 hpf embryos exhibited the decreased number of PGCs with the weaker fluorescence (**B**). The number of PGCs was fewer and the fluorescent signal became weaker in the MTZ-treated embryos at 36 hpf (**D**). At 48 hpf, the fluorescence labeled-PGCs almost disappeared (**F**). No fluorescence labeled-PGCs were observed in the MTZ-treated embryos at neither 60 hpf (**H**) nor 72 hpf (**J**).

After treating the embryos derived from the female transgenic zebrafish of Tg(*nanos3*:nfsB-mCherry-nanos3 3′UTR) with 10 mM MTZ for 48 h from 0 hpf through 48 hpf (referring as MTZ-treated embryos hereafter), we examined the number and distribution of PGCs under fluorescence microscopy. When observed at 24 hpf, the control embryos without MTZ treatment (referring as control embryos hereafter) displayed most labeled PGCs colonizing the genital ridge (Fig. 2A) whereas the MTZ-treated embryos had obviously fewer PGCs colonizing in the genital ridge and some labeled cells were incomplete migration towards the genital ridge (Fig. 2B). Throughout development, the most of labeled cells were arranged around the genital ridge in the control embryos (Fig. 2C) whereas fewer cells were seen in the genital ridge and the labeled cells were incomplete migration towards the genital ridge of the MTZ-treated embryos at 36 hpf (Fig. 2D). When observed at 48 hpf, almost no fluorescent cells can be seen in the MTZ-treated embryos (Fig. 2F) while the PGCs labeled by mCherry fluorescent were rich around the gential ridge of the control transgenic embryos (Fig. 2E). By 60 hpf, the fluorescent intensity of PGCs was slightly reduced and dispersed in the control embryos (Fig. 2G), and this became more evident at 72 hpf (Fig. 2I). However, no fluorescent cells or PGCs were found in the MTZ-treated embryos at neither 60 hpf (Fig. 2H) nor 72 hpf (Fig. 2J). Taken together, the results suggest that MTZ treatment not only affected early migration but also targeted cells for elimination of the PGCs.

### No germ cells are present in the gonads of 20-dpf juvenile developed from the MTZ-treated transgenic zebrafish embryos

**Figure 3:**
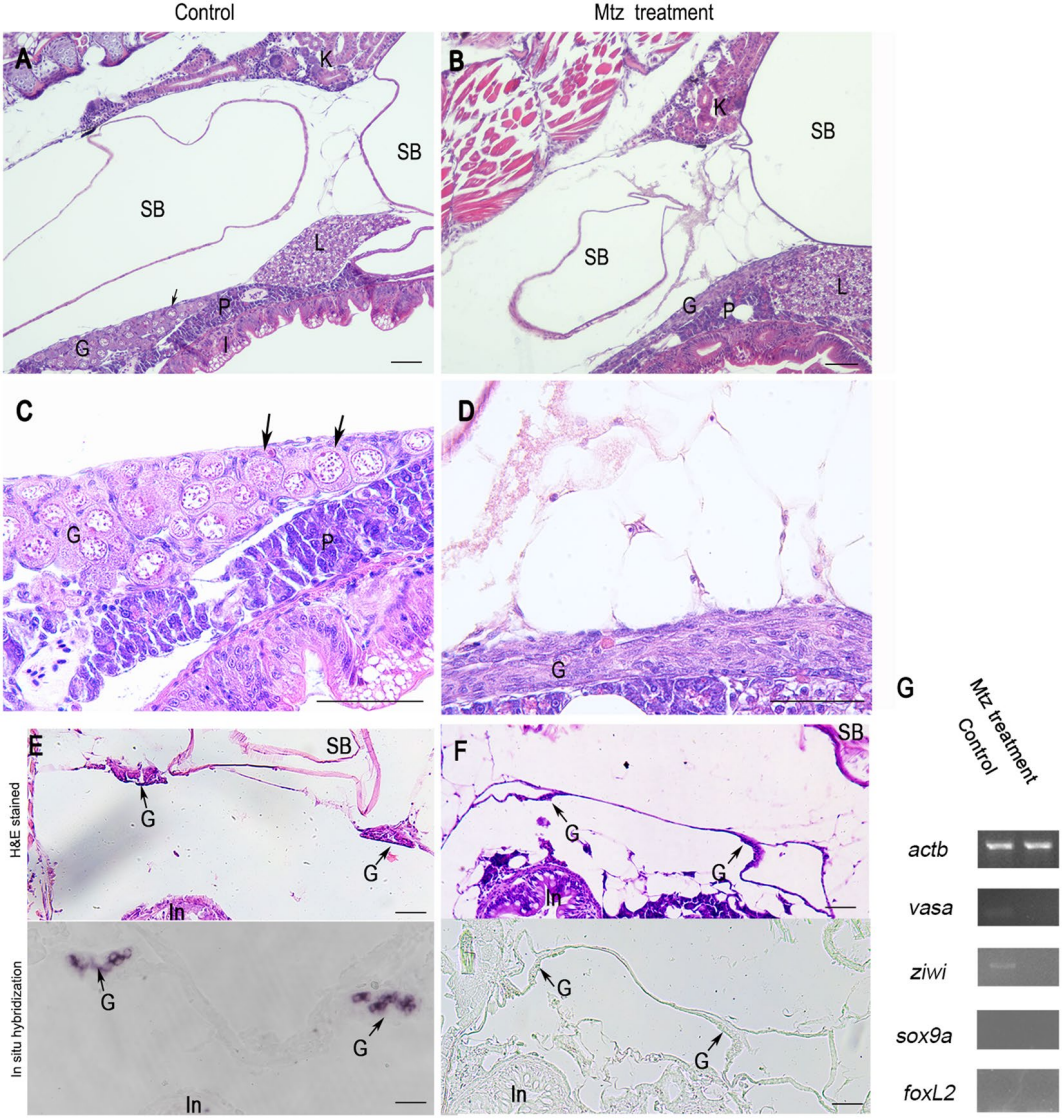
Germ cells ablation in 20-dpf zebrafish developed from MTZ-treated embryos. Longitudinal sections showing that the gonads of control zebrafish at 20-dpf (**A** and **C**) carried a large number of early perinucleolar oocytes (black arrow) with associated somatic tissue, whereas MTZ-treated zebrafish at 20-dpf had only somatic gonadal cells (**B** and **D**). Transverse sections showing that the gonads of control zebrafish at 20-dpf (**E**, upper panel) carried normal gonads (black arrow) with associated somatic tissue and had noraml *vasa* expression in gonads (**E**, lower panel, black arrow) detected by *in situ* hybridization, whereas the gonads of MTZ-treated zebrafish at 20-dpf had only somatic gonadal cells (**F**, upper panel) and had no *vasa* expression (**F**, lower panel) detected by *in situ* hybridization. (**G**) RT-PCR results showing the weak expressions of *vasa* and *ziwi* were detected in the gonads of control zebrafish but not in the zebrafish developed from the MTZ-treated embryos, and no expressions of *sox9a* and *foxL2* was detetced in neither control zebrafish nor MTZ-treated zebrafish. Expression of *actb* was used as positive control. K: kidney, SB: swim bladder, G: gonad, L: liver, P: pancreas, I: Intestine. Scale bars = 20 µm (**A**,**B**,**C**,**D**) and 40 µm (**E**,**F**).

The differentiation and development of gonad in zebrafish are relatively distinctive during early stage. Both male and female zebrafish undergo a juvenile ovary stage and subsequently proceed into the gonadal differentiation stage^21^. Histological examination of H&E-stained sections showed that the gonadal tissues of 20-dpf juvenile derived from control embryos mainly localized in the upper posterior cavity, specifically nearby the posterior chamber of the swim bladder, the hepatopancreas, and intestinal tract (Fig. 3A), where numerous perinucleolar oocytes in the gonad indicated the juvenile ovary stage (Fig. 3C). However, the 20-dpf juvenile derived from the MTZ-treated embryos displayed a gonad-like structure beneath the swim bladder (Fig. 3B), primarily consisting of supporting cells with no detectable perinucleolar early oocytes or other germ cells (Fig. 3D). Thus, MTZ treatment appears to completely eliminate the germ cells in the juvenile zebrafish developed from the female transgenic zebrafish Tg(*nanos3*:nfsB-mCherry-nanos3 3′UTR), while somatic cells adjacent to the gonadal tissues normally differentiates into supporting structures in the absence of germ cells.

To confirm the above observation, we performed *in situ* hybridization on the sections of gonad with the antisense RNA probe of *vasa*. The results showed that no *vasa* positive cells were found in the gonad tisssues of the 20-dpf juvenile developed from MTZ-treated embryos (Fig. 3F) while the *vasa* expression was obviously present in the gonad of the juvenile derived from the control embryos (Fig. 3E). Morever, results from RT-PCR analysis revealed that the 20-dpf juvenile derived from MTZ-treated embryos exibited no expressions of *vasa* and *ziwi* that were normally expressed in the juvenile developed from control embryos (Fig. 3G). And the 20-dpf juvenile neither from MTZ-treated embryos nor from control embryos had the expressions of *sox9a* or *foxl2* (Fig. 3G). The results suggest that no germ cells were present in the 20-dpf juvenile developed from MTZ-treated embryos.

### Adult transgenic zebrafish derived from MTZ-treated embryos exhibit male-like morphology

**Figure 4:**
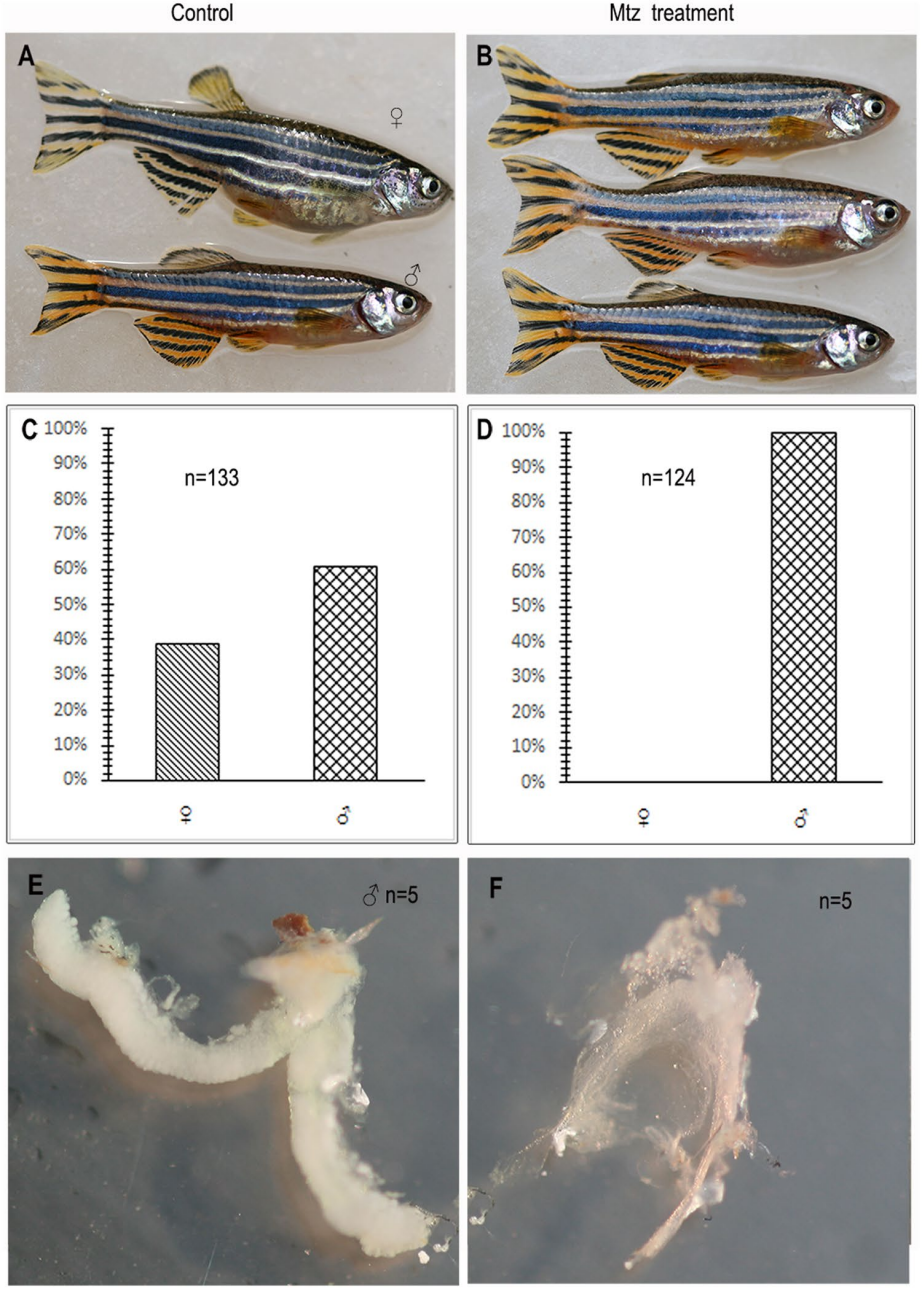
MTZ-treated embryos develop into male-like adult zebrafish with shrunken testis 100-dpf adult zebrafish developed from control or MTZ-treated embryos. Both male and female zebrafish were found in the control (**A**), while all the adult zebrafish derived from the embryos treated with MTZ (**B**) were male-like with typical sexually pigmentation. The female/male ratio in control (**C**) or MTZ treatment group (**D**) were also analyzed. Dissected the testis from adult control and MTZ-treated zebrafish revealed that the testis of treated fish were shrunken, significantly smaller than those of controls (**E**,**F**).

The appearances of male and female adult zebrafish differ substantially. Females are plumper than males due to expansion of the abdomen with ovarian development. In contrast, males are longer and slender with larger fins, especially the anal fin, than females. Sexually mature males are bright yellow-brown (the normal nuptial color), especially in the anal region and caudal fin. The abdominal regions adjacent to each fin are also brightly colored (Fig. 4A), especially at daybreak. Mature males are observed chasing females counterparts, a normal mating behavior. Interstingly, the adult zebrafish derived from MTZ-treated embryos exhibited no observable disparities in feeding, behavior, and growth. The majority of them looked long and slender at 60 dpf, and displayed obvious male characteristics (Data not shown). Morever, no evident abdominal expansion was observed in 124 of the adult zebrafish derived from the MTZ-treated embryos. They all displayed the normal nuptial color (Fig. 4B,D). In contrast, about 39% (52 of 133) of the adult zebrafish derived from the control embryos appeared to be females and the other 61% (81 of 133) exhibited male morphology (Fig. 4A,C) when reaching 100 dpf.

### Effect of MTZ treatment on reproductive capability

The adult zebrafish derived from the MTZ-treated embryo showed normal mating behavior (chasing), and stimulated females to lay eggs. However, at approximately 4 h after spawning, the eggs, prusumbly fertilized by 10 of the different adult zebrafish with male apperances, all gradually became white and died. In the control group, the eggs fertilized by the adult zebrafish derived from control embryos developed normally, and the hatching rate all exceeded 90%. The results demonstrate that the eggs were not able to be fertilized by the adult zebrafish derived from the MTZ-treated embryos though they exhibited male-like morphology and normal reproductive behaviors.

### Effect of MTZ on the gonad structure of mature zebrafish

The testes of the sexually mature male zebrafish locate bilaterally at the dorsal side of the digestive tract, closely adhered to the abdominal wall and the ventral side of the swim bladder, occasionally surrounded by adipose tissues. Both cylinder-shaped testes extend from the middle abdominal cavity to the anal fin, converging to form a V shape at the end of the posterior abdominal cavity with separation at the gonopore. Bilateral gonads were fully developed in the adult zebrafish derived from the control embryos, and were cream white in color and plump in shape (Fig. 4E). The gonadal position of the adult zebrafish derived from MTZ-treated embryos was similar to that of control males, and their gonadal tissues still formed a bilateral structure. Additionally, their general shape of the testis was preserved, and bilateral gonadal tissues also converged to the gonopore. However, after removal of the adipose tissues, the gonadal tissues appeared completely withered and shrunken (Fig. 4F) compared to testes of the adult zebrafish developed from the control embryos (Fig. 4E).

**Figure 5:**
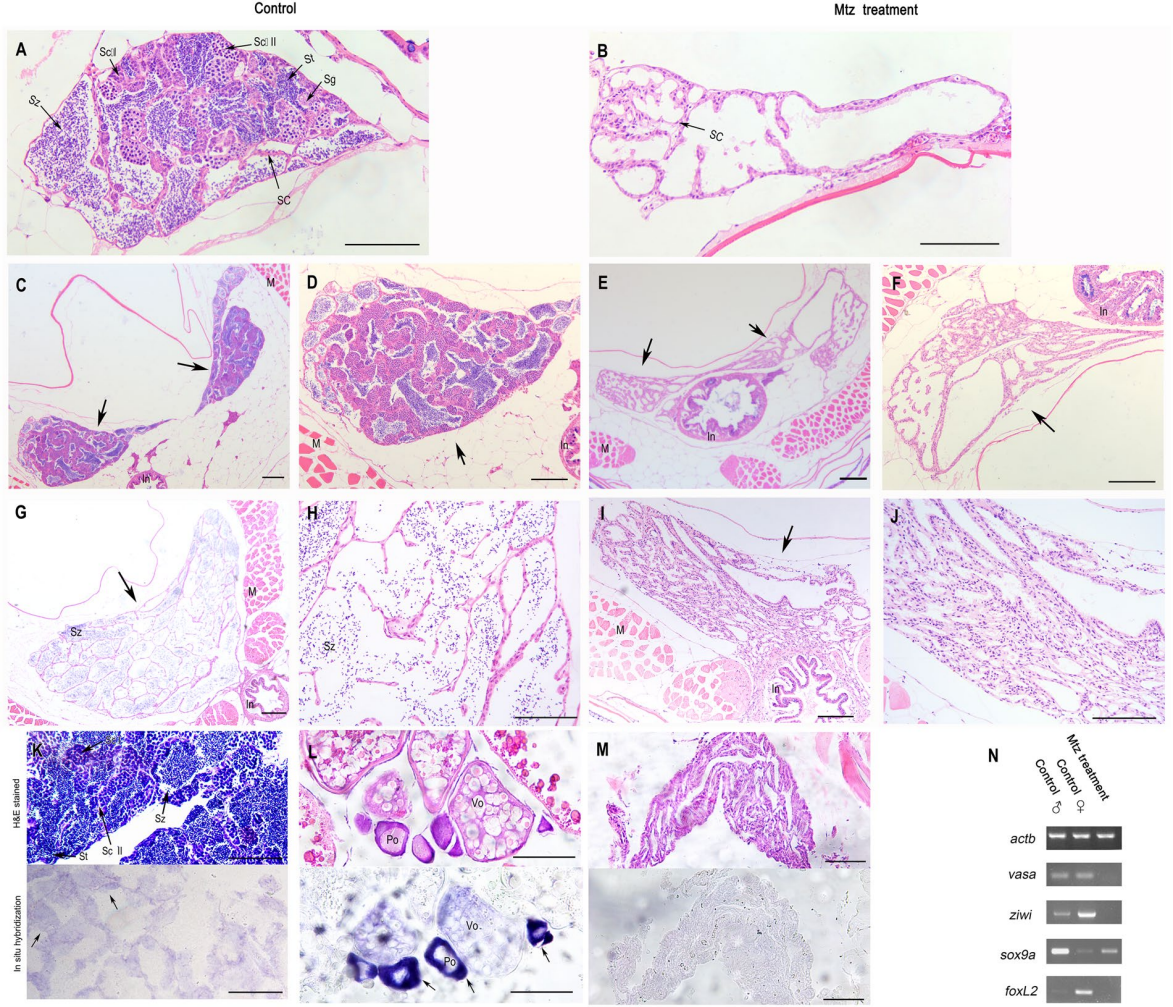
Histological analysis revealed the absence of germ cells at various developmental phases in the testis of MTZ-treated adult zebrafish. Transverse sections showing that the gonads of control zebrafish (**A**,**C**,**D**,**G**,**H**) and the ones of MTZ-treated zebrafish (**B**,**E**,**F**,**I**,**J**) at 100-dpf. At top of bilateral testis in the control adult zebrafish, germ cells with various developmental phases were found: spermatogonia, primary spermatocytes ScI, secondary speratocytes ScII, spermatids and spermatozoa (**A**), whereas MTZ-treated zebrafishes were found to have only Sertoli cells which were derived from somatic cells (**B**). Testis (arrow) near the bilateral gonadal junction also carried numerous seminiferous tubules with irregular shape in control adult fish (**C**,**D**), but there was no germ cells in the gonad (arrow) of the MTZ-treated fish at any developmental phases observed (**E**,**F**, arrow). Seminal vesicle-like structures were found in cross-section of the testis adjacent to the gonopore. The organ (arrow) consisted of numerous cavities communicated with each other, that contained a large quantity of sperm cells in the control zebrafish (**G**,**H**). No sperm was detected within the seminal vesicle in gonads (arrow) of the MTZ-treated zebrafish, so the cells of cavities which derived from somatic cells formed net structures (**I**,**J**). Transverse sections showing that the gonads of control male zebrafish at 100-dpf (**K**, upper panel) carried normal testis (black arrow) with germ cells at different developmental phases and had weak *vasa* expression in gonads (**K**, lower panel, black arrow) detected by *in situ* hybridization, the gonads of control female zebrafish at 100-dpf (**L**, upper panel) carried normal ovary and had strong *vasa* expression in developing oocytes (**L**, lower panel, black arrow) detected by *in situ* hybridization, whereas the gonads of MTZ-treated zebrafish at 100-dpf had only somatic gonadal cells (**M**, upper panel) and had no *vasa* expression (**F**, lower panel) detected by *in situ* hybridization. (**N**) RT-PCR results showing the expressions of *vasa* and *ziwi* were detected in the gonads of control male and female zebrafish, but not in the zebrafish developed from the MTZ-treated embryos, and strong expressions of *sox9a* and *foxl2* were detetced in control male zebrafish and control female zebrafish respectively, weak expression of *sox9a* was found in control female zebrafish and MTZ-treated zebrafish, and no expression of *foxl2* was detetced in control male zebrafish and MTZ-treated zebrafish. Expression of *actb* was used as positive control. Sg: spermatogonia, Sc I: Primary spermatocytes, Sc II: Secondary spermatocytes, St: Spermatids, Sz: Spermatozoa, SC: Sertoli cell, In: Intestine, M: Muscle, K: Kidney, Po: Primary oocyte, Vo: Vitellogenic oocyte. Scale bars = 20 µm.

The fine structural features of mature zebrafish testes were then examined in thin sections throughout the transverse axis (Table 1). In the adult zebrafish developed from the control embryos, the testis was likely divided into two functional parts including bilateral tetses and intersection point. Bilateral testes are responsible for sperm production. They were surrounded by membrane structures and both consisted of a large quantity of irregularly shaped seminiferous tubules, which were interleaved and intimately arranged. Cross sections of seminal vesicles, the central structure of the seminiferous tubule, were generally circular or oval. The seminal vesicles consisting of germ cells were enveloped by Sertoli cells. Sperm were formed within the seminal vesicles, and the germ cells within it were derived from the same spermatogonia with nearly synchronous development. However, the germ cells between adjacent seminal vesicles differed in developmental phases. Spermatogonia, primary spermatocytes, Sc I, secondary speratocytes, Sc II, spermatids, and spermatozoa could be observed within a single cross section (Fig. 5A,C,D). In contrast, the adult zebrafish developed from MTZ-treated embryos exhibited neither germ cells at any developmental phases nor seminiferous tubule-like structures. Rather, the gonadal structure was composed of grid-shaped supporting tissues consisting of Sertoli cells and connective tissues (Fig. 5B,E,F). Sertoli cells were seen in multi-porous structures due to the lack of filling by functional sperm cells, consistent with the gross withering and shrinkage of the gonad.

**Table 1:** Summary of the histological results for different types of cells detected in adult zebrafish testis.

	Cells detected						
	Numbers of fish	Spermatogonia	Primary spermatocyte	Secondary spermatocyte	Spermatid	Spermatozoa	Sertoli cell
Control	10	10	10	10	10	10	10
MTZ treatment	10	0	0	0	0	0	10

In the adult zebrafish developed from control embryos; however, the testes intersection point stored the sperm. It consisted mainly of seminal vesicle-like structures distinct from those of bilateral testes. No spermatogonia or spermatids were seen; rather, the structure consisted of a large quantity of honeycomb-shaped tissues composed of multiple irregular cavities forming ridge structures. These cavities were full of sperm, similar to functional seminal vesicles (Fig. 5G,H). We proposed that the sperm were generated in the seminiferous tubules and transferred from the seminal duct for temporary storage. Cross-sections close to the intersection point also revealed long strips of tissue in a dumbbell-shape. There was no significant change in the structures adjacent to the cloacel opening, whereas the cross-sections gradually became round, consistent with the overall appearance of the testes at this position (Fig. 5G,H). The gonadal structures of the adult zebrafish developed from the MTZ-treated embryos were similar to those of controls, but no sperm were detected within the seminal vesicles. Cavities consisting of Sertoli cells were seen, which subsequently formed network structures (Fig. 5I,J). In the adult zebrafish developed from control embryos, the seminal vesicles were full of semen to nourish the sperm and maintain ionic equilibrium (Fig. 5G,H). In the adult zebrafish developed from MTZ-treated embryos, however, the testis lacked germ cells, failed to produce and store the sperm, and were withered, all manifested as collapsed network structures under the microscope (Fig. 5I,J).

To confirm the histological observation, we performed *in situ* hybridization on the sections of gonads with the antisense RNA probe of *vasa*. The results revealed that no *vasa* positive cells were found in the gonad tisssues of adult zebrafish (100-dpf) derived from MTZ-treated embryos (Fig. 5M) while the *vasa* expressions were obviously present in the primary spermatocytes (Sc I) and secondary speratocytes (Sc II) of the testis in the adult male zebrafish derived from control embryos (Fig. 5K), and in the primary oocyte (Po) of the ovary in the adult female zebrafish derived from control embryos (Fig. 5L). Morever, results from RT-PCR analysis showed that the gonads of 100-dpf adult zebrafish developed from MTZ-treated embryos had no expressions of *vasa* and *ziwi* that are normally expressed in the ovary or tetsis in the adult zebrafish derived from control embryos (Fig. 5N). Consistently, *foxl2*^22,23^, expressed in ovary but not in testis of the adult zebrafish developed from the control embryos, was not found to express in the gonads of adult zebrafish developed from the MTZ-treated embryos. In contrast, *sox9a*, strongly expressed in testis^22^ but weakly in ovary of the adult zebrafish developed from the control embryos, was also found to express weakly in the gonads of adult zebrafish developed from the MTZ-treated embryos (Fig. 5N). Taken together, the adult zebrafish developed from the MTZ-treated embryos had no germ cells at all.

## Discussion

Fish gonads are derived from two types of cells: PGCs and somatic cells adjacent to the genital ridge. PGCs are critical determinants of zebrafish sex as they can differentiate into either ovogonium or spermatogonia. During early embryonic development, PGCs aggregate at a specific position, undergo oriented migration to the gonadal primordium, and develop with surrounding somatic-derived cells to form a functional gonad where PGCs differentiate into two types of germ cells namely sperm and oocyte, and somatic cells adjacent to germinal primordium develop into the structures supporting the gonadal tissues^24^.

The NTR/MTZ system can specifically eliminate cells or tissues, and currently serves as an effective approach for investigating the mechanism of organ function and regeneration^11,25^. Previously, Hsu *et al*. generated transgenic zebrafish Tg(*asp*:nsfB), Tg(*odf*:nsfB) and Tg(*sam*:nsfB) in which NsfB was expressed specifically in testis under the promoter of *asp* (A-kinase anchoring protein-associated protein), *odf* (outer dense fibers) and s*am* (sperm acrosomal membrane-associated protein)^14^. After treated with MTZ, 68%, 59%, 54% of the transgenic zebrafish displayed male sterile, respectively. Because *asp*, *odf* and *sam* were all expressed in spermatid or sperm, the transgenic zebrafish treated with MTZ still had spermatogonia in their testis although the spermatid was ablated by MTZ treatment. Therefore, it is reasonable to hypothesize that the normal sperm can be developed and the transgenic zebrafish are fertile again once MTZ is removed because the spermatogonia are not affected by MTZ at all. In contrast, two other groups^25,26^ generated transgenic zebrafish Tg(*zp*:nsfB) in which the expression of *nsfB* is specifically driven by the promoter of ovarian specific expression gene of *zp* (zona pellucida). When treated with MTZ, the transgenic zebrafish exhibited female sterile because the disrupted formation of zona pellucida blocked the oogenesis although oogonium were not affected at all. Once MTZ was removed, oogenesis was recovered and normal oocyte was developed from the unaffected oogonium, resulting in the female transgenic zebrafish fertile.

Unlike sperm and oocyte that are formed in testis and ovary respectively, PGCs are the germ stem cells that are specified in the embryos at early development and are fate to differentiate into gametes. Therefore, the fish would be sterile if their PGCs are eliminated in the embryos at early development. Actulaly, in the absence of PGCs, the somatic cells of zebrafish and medaka (*Oryzias latipes*) gonads tend to spontaneously differentiate into male characteristics, while gonadal differentiation with female characteristics requires PGCs^15,27–31^, although the underlying mechanisms remain to be elucidated. Consistenly, Wong and Collodi reported that overexpressing stromal-derived factor 1a (Sdf1a), providing the directional cue that guides the migration of PGCs to the gonadal tissue in a gradient, disrupted the normal PGC migration pattern, resulting that the embryos developed into sterile males^9^. Additionally, microinjection of *dnd* morpholino into zebrafish fertilized eggs interfered with the migration and formation of PGCs, resulting in zebrafish with male characteristics^22^. In this study, we set up a transgenic zebrafish line Tg(*nanos3*:nfsB-mCherry-*nanos3* 3′UTR). Driven by *nanos3* promoter, the transgene was expressed during oogenesis but not spermatogenesis, and the mRNA message was deposited in oocyte. After fertilization, the message in the oocyte was alloted into PGCs but not somatic cells because of the 3′UTR of *nanos3* that allows the mRNA be stable in PGCs but be degradated in somatic cells^32^. In other words, only the PGC cells in the embryos derived from female transgenic zebrafish can express the fusion protein of NfsB-mCherry. Therfore, once the embryos are incubated with MTZ, the PGC cells in the embryos derived from the female transgenic zebrafish will be killed no matter the embryos themselves are transgenic or not. Conforming to the above hypothesis, our results demonstrated that early MTZ treatment completely eliminated PGCs in the embryos derived from the female transgenic zebrafish. With development, no germ cells were detected in the gonads of the zebrafish developed from the MTZ-treated embryos (Fig. 3), and only cavity-shaped structures derived from somatic cells were observed (Fig. 4), resulting in all male-like sterile adults without reproductive function (Figs 4 and 5).

As an anti-anaerobe and protozoacide, prolonged treatment with high-dose MTZ may induce adverse effects^33^. Therefore, the duration and dose of MTZ treatment are the key parameters for targeted conditional elimination. Previously, Curado *et al*. utilized 10 mmol/L MTZ to treat transgenic NTR juvenile zebrafish under the control of different promoters and found that cell ablation occurred in the heart, pancreas, and liver at 24 h, accompanied by functional defects^34^. White *et al*. adopted 5 mmol/L MTZ to treat transgenic animals and conditionally removed the ovary^26^. Hsu *et al*. treated transgenic zebrafish using 5 mmol/L MTZ for 14 d and demonstrated that the testis was completely or partially destroyed^14^. In this study, we demonstrated that a dose of 10 mmol/L Mtz for 48 h from 1-cell stage exerted no significant adverse effects on zebrafishembryos and all the embryos treated with Mtz developed into male-like sterile adult. The results revelaed that short-term MTZ intervention with 10 mM is sufficient to effectively eliminate germ celles at early development. However, 5 mM is not optimal amount to kill all the germ cells in zebrafish embryos because Dai *et al*. administrated 5 mM MTZ to treat transgenic zebrafish Tg(*dnd*:NTR-EGFP + 3′UTR) for 20 days from 18 dpf and found that all MTZ-treated transgenic fish exclusively developed into males with subfertilities^15^.

In aquaculture, males of multiple species, such as tilapia (*Oreochromis niloticus*) and yellow catfish (*Tachysurus Fulvidraco*), outperform females in terms of breeding and growth. For example, if male and female tilapia are raised together, they reach sexually mature at the age of 3–5 months and they then mate *ad libitum* to produce offspring. The adults derived from the offspring are in small body size, far less than the market size. Therefore, production of all-male fish may substantially improve aquaculture yield of the defined fish species^35,36^. In this study, we found no germ cells were detected in the gonads of MTZ-treated zebrafish at 20 dpf. In addition, no PGCs-derived germ cells except somatic cell-derived supporting structures were observed in the gonads of MTZ-treated transgenic zebrafish. Interestingly, the MTZ-treated adult zebrafish retained the morphology and behavioral characteristics of males, but failed to produce sperm despite chasing and attempting to mate with females for spawning. The results demonstrate that zebrafish with conditional elimination of PGCs at early development grow up as sterile male-like fish. Therefore, the NTR/MTZ system established to conditionally eliminate PGCs in this study provides an alternative novel strategy for producing all-male fish, which is probably a practical direction for fishery industrialization. Provided that the female transgenic fish is a heterzygote for the transgene, only half of their offspring will be transgenic and the other half will be non-trangenic but their germ cells were all killed when they are incubated with MTZ. Therefore, one can obtian non-trangenic all male-like fish in aquaculture industry using this developed techanique system.

Transplantation of fish germ cells offers a novel strategy for rapid breeding and seed conservation^37,38^, but this strategy is unfeasible for industrialized application due to low transplantation efficiency (which may be enhanced depending on the donor fish cells and the quality of the recipient fish). Currently, multiple recipient fish species lack a sterile line^39,40^. Conventionally physical and chemical approaches have been employed, but they have either failed to completely eliminate the germ cells or resulted in substantial variability of outcome, which greatly increases the workload for subsequent screening. Furthermore, these non-targeted methods can alter other tissues^2^. Thus, the experimental endpoint cannot be controlled and reproducibility is poor. In this study, the germ cells of adult zebrafish developed from the MTZ-treated embryos were completely eliminated, which can avert the germ cell chimeras between the donor and recipient fish and facilitate subsequent screening. Alternatively, the somatic cell-derived supporting structures remain in intact, thereby creating a favorable environment for the development and differentiation of donor cells and enhancing transplantation efficiency. Thus, induced elimination of germ cells as demonstrated in this study may facilitate the systematic development of appropriate recipient fish for the transplantation of allogenic germ cells.

## Experimental Methods

### Ethics statement

Experimental protocols using zebrafish as a research subject in this study were approved by the Aquatic Animal Research Committee at Pearl River Fishery Research Institute, China. All methods were performed in accordance with the relevant guidelines and regulations.

### Generation of transgenic zebrafish

A Tol2-nanos3-nfsB-mCherry-nano3-3′UTR vector (Fig. 1A) was constructed using traditional molecular recombination. In the expression construct of Tol2^41^, the CDS of nfsB-mCherry, encoding a fusion protein of NfsB nitroreductase fused with mCherry reporter, was inserted between the promoter of zebrafish *nano3* and its 3′UTR^40^ in order for the transcription of the fused gene to be controlled by zebrafish *nano3* promoter and the transcript to be only stably present in PGCs^42^. A transgenic line was established by microinjecting the transgenic construct plus transponase mRNA into zebrafish fertilized eggs as we described previously^43^. F0 zebrafish were bred to sexual maturity, and the females selected and mated with wild-type males to produce F1 progeny. The transgenic F1 was determined by the strong occurrence of mCherry fluorescence in PGCs at 24 hpf under fluorescence microscopy. The number of fluorescence-labeled embryos derived from per female was recorded and embryos without fluorescence were abandoned. At 2 months of age, the caudal fin of fluorescence-labeled zebrafish (F1) was clipped for the template preparation of genomic DNA using a commercial kit (YSY, China). PCR amplification was performed using *nfsB* gene-specific primers (CATTCCACTAAGGCATTTGA and GTTTTGCGGCAGACGAGATT) and the PCR products were characterized by agarose gel (1%) electrophoresis and Sanger sequencing. F1 Zebrafish carrying the *nfsB* gene were used for strain breeding and subsequent analysis.

### Transgene copy number determination

Quanntative PCR was performed using the equipment of ABI 7500. To determine the copy numbe of transgene in the genome of trangenic zebrafish, two standarad curves (log of copy number vs amplification cycle threshold)^19,20^, one for endogenous *actb* and the other for trangene *nfsb*, were first set up by the real time PCR using the pair of primers of ACGAACGACCAACCTAAACTCT and TTAGACAACTACCTCCCTTTGC (*actb*), and CATCCAGCACCAACTCCCA and CCAGCTTCAGCCAGACATCGT (*nsfB*), respectively. The standard template of *actb* DNA sample was the genome DNA isolated from the caudal fin of wild type zebrafish using the tradiational genomic DNA isolation mthod. In contrast, the standard template of *nsfB* DNA sample was the cloning vector of the transgene. The copy number of target template was calcualted using the equations of DNA concentration (ng/μL) × 10^−6^ × 1 μL × 10^−3^ × 6.02 × 10^23^/(6593 × 650) (for transgene *nsfb*) or DNA concentration (ng/μL) × 10^−6^ × 1 μL × 10^−3^ × 6.02 × 10^23^/(1.7 × 10^9^ × 650) (for *actb*). The PCR reaction was performed in a 20 μL volume including 10 μL of SYBR Premix Ex Taq II (2×), 0.8 μL of 10 μM forward primer, 0.8 μL of 10 μM reverse primer, 0.4 μL of ROX Refernce Dye II (50×), 6 μL of ddH_2_O, and 2 μL of standard DNA template. The PCR was run in 95 °C 30 s, followed by 40 cycles of 95 °C 5 s, 60 °C 34 s, and 72 °C 15 s. Each amplification was repeated 3 times. The cuve was plotted with X axis representing log of copy number and Y denoting the average of Ct.

To determine the copy nummber of the transgene in the transgenic zebrafish genome, genomic DNA was isolated from the caudal fin of the transgenic zebrafish as descibed above and then used as the template of the quantative PCR amplifying the trangene and the endogenous *actb* gene. The copy number of trangene was finnally calcualated from the standard curve by comparing it with *actb* that are two copies in the zebrafish genome.

### MTZ treatment

Female zebrafish with positive phenotype (mCherry fluorescence) and genotype (*nfsb*) were mated with wild type male and the fertilized eggs collected. Metronidazole (MTZ) was dissolved in DMSO and adjusted to 10 mM with water. Sixty mCherry-positive fertilized eggs were obtained and treated with 10 mM MTZ in a 500 mL beaker at 28 ± 1 °C under darkness. After 24 h, the medium was exchanged with fresh MTZ. After another 24 h, the solution was exchanged for egg water and incubation was continued. Growth and development status were monitored regularly hereafter. All treatments were conducted in triplicate.

### Effect of MTZ treatment on PGC morphology

After 24, 36, 48, 60 and 72 h of MTZ treatment, four embryos per group were randomly selected and PGC morphology and distribution assessed under fluorescence stereoscopy.

### Effect of MTZ treatment on gross appearance of zebrafish

Zebrafish body and color changes were observed at each time point and compared between the MTZ treatment and control groups.

### Effect of MTZ treatment on gonadal tissues of 20-dpf zebrafish

Ten zebrafish were randomly selected after 20 dpf (days post fertilization) of MTZ treatment and fixed in 4% paraformaldehyde solution overnight at 4 °C. The juvenile were then dehydrated in gradient ethanol, made translucent by xylene treatment, embedded by paraffin, sectioned at 3 μm longitudinally along the body axis or transversely, stained with hematoxylin and eosin (H&E), and finally mounted. The histology of gonadal tissues was compared between MTZ-treated and control groups under light microscopy.

### Assessment and validation of reproductive capability

Ten of 100-dpf zebrafish developed from the MTZ-treated embryos were randomly selected and mated with wild-type female zebrafish, and another 10 transgenic male zebrafish were chosen as normal controls. Spawning and reproduction status were compared between the two groups.

### Gross observation of gonad tissues of adult zebrafish

Five of 100-dpf zebrafish developed from the MTZ-treated embryos or control males were randomly chosen and sacrificed under anesthesia using 0.2 mg/ml Tricaine. The size and morphology of the zebrafish gonad were compared between MTZ-treated and control transgenic males. Differences in morphology and positions of liver, intestine, kidney, and other organs were also examined to evaluate extraneous effects of MTZ.

### Comparison of gonadal histology in adult zebrafish

Ten of 100-dpf zebrafish developed from the MTZ-treated embryos and control males were randomly chosen, fixed, decalcified for 2 weeks, transverse sectioned, and stained with H&E. The gonadal structure and germ cellular morphology were compared.

### RT-PCR

Gonads were isolated from the zebrafish developed from MTZ-treated embryos or control embryos without MTZ treatment at 20 dpf and 100 dpf under the stereoscope. Samples were homogenized with a mortar and pestle in liquid nitrogen. Total RNA was then extracted with TRIzol Reagent (Invitrogen) according to the manufacturer’s instructions. The quality of the extracted RNA was determined by agarose gel electrophoresis. cDNA was reverse transcribed using the TAKARA PrimeScript™ RT reagent Kit with gDNA Eraser (TAKARA, Japan). cDNA was stored at −20 °C for gene expression analysis.

PCR reactions were performed in 25 μl volume containing 9.5 μl ddH_2_O, 1 μl cDNA template, 1 μl (10 μM) of each primer, 12.5 μl 2 × Taq PCR MasterMix (TianGen, China). The seqeucnes of the forward and reverse primers used to detect gene expressions were CCCAATATGGATGACTGGGAG and GTCATTTTCCATGAGCTACC (for *vasa*), CTCAGATGGTGGTGGTGATCT and ACGGTCACACTGTTCCTTCAG (for *ziwi*), AGTCCACACGTTTCCTGATTG and ATCCTGTGGAATTCTGTGACG (for sox9a), CCCAGCATGGTGAACTCTTAC and CGTGATCCCAATATGAGCAGT (for *foxl2*), GAGAAGATCTGGCATCACACCTTC and GGTCTCGTGGATACCGCAAGATTC (for *actb*). The PCR was run in 95 °C 5 min, followed by 35 cylces of 95 °C 30 s, 60 °C 30 s and 72 °C 30 s, and finally extended for 5 min at 72 °C. The PCR products was seperated by 1.0% native agarose gel electrophoresis.

### *In situ* hybridization

Gonads were isolated from the zebrafish developed from MTZ-treated embryos or control embryos without MTZ treatment at 20 dpf and 100 dpf under the stereoscope. They were fixed in 4% paraformaldehyde solution overnight at 4 °C, dehydrated and embedded as described above, then sectioned at 7 μm transversely. Some sections stained with H&E and others for hybridizations. A 1223 bp *vasa* cDNA fragment was inserted into a pGEM-T vector for the synthesis of antisense RNA probes under the drive of T7 promoter by using the digoxigenin RNA Labeling Kit (Roche). The RNA probes were treated with RNase-free TURBO DNase and purified with SigmaSpinTM Sequencing Reaction Clean-Up (Sigma). The sections were digested with proteinase K (10 μg/ml) for 10 min and hybridized with the probes at 65 °C for 14 hours. Chemical *in situ* hybridization was conducted by developing the signals with BCIP/NBT substrates on sections and post fixed in 50% glycerin. The results was photopgraphed under microsope uisng digital CCD camera.

## Electronic supplementary material

Supplementary information

## References

[1] Zhang Y (2015). A controllable on-off strategy for the reproductive containment of fish. Sci Rep..

[2] Golpour A, Siddique MA, Siqueira-Silva DH, Pšenička M (2016). Induced sterility in fish and its potential and challenges for aquaculture and germ cell transplantation technology: a review. Biologia.

[3] Manor ML, Weber GM, Cleveland BM, Kenney PB (2014). Effects of feeding level and sexual maturation on fatty acid composition of energy stores in diploid and triploid rainbow trout (*Oncorhynchus mykiss*). Aquaculture.

[4] Gui J, Zhou L (2010). Genetic basis and breeding application of clonal diversity and dual reproduction modes in polyploid *Carassius auratus* gibelio. Sci China Life Sci..

[5] Li Y (2015). Meiotic chromosome configurations in triploid progeny from reciprocal crosses between wild-type diploid and natural tetraploid loach *Misgurnus anguillicaudatus* in China. Genetica.

[6] Richardson BE, Lehmann R (2010). Mechanisms guiding primordial germ cell migration: strategies from different organisms. Nat Rev Mol Cell Biol..

[7] Lauth, X. & Buchanan, J. T. In US Patent & Trademark Office, Patent Full Text and Image Database. (ed. U.P.T. Office) (AquaBounty Technologies, Inc. (Waltham, MA) United State; 2012).

[8] Wong TT, Zohar Y (2015). Production of reproductively sterile fish: A mini-review of germ cell elimination technologies. Gen Comp Endocr..

[9] Wong TT (2013). Inducible sterilization of zebrafish by disruption of primordial germ cell migration. Plos one.

[10] Curado S, Stainier DY, Anderson RM (2008). Nitroreductase-mediated cell/tissue ablation in zebrafish: a spatially and temporally controlled ablation method with applications in developmental and regeneration studies. Nat Protoc..

[11] Zhang R (2013). *In vivo* cardiac reprogramming contributes to zebrafish heart regeneration. Nature.

[12] He J, Lu H, Zou Q, Luo L (2014). Regeneration of liver after extreme hepatocyte loss occurs mainly via biliary transdifferentiation in zebrafish. Gastroenterology.

[13] Pisharath H (2007). Targeted ablation of beta cells in the embryonic zebrafish pancreas using *E. coli* nitroreductase. Mech Develop..

[14] Hsu CC (2010). Inducible male infertility by targeted cell ablation in zebrafish testis. Mar Biotechnol..

[15] Dai X (2015). Sufficient numbers of early germ cells are essential for female sex development in Zebrafish. PloS one.

[16] Hartung O, Forbes MM, Marlow FL (2014). Zebrafish *vasa* is required for germ cell differentiation and maintenance. Mol Reprod Dev..

[17] Houwing S (2007). A role for *Piwi* and piRNAs in germ cell maintenance and transposon silencing in zebrafish. Cell.

[18] Li M (2016). Dazl is a critical player for primordial germ cell formation in medaka. Sci Rep..

[19] Ballester M, Castello A, Ibanez E, Sanchez A, Folch M (2004). Real-time quantitative PCR-based system for determining transgene copy number in transgenic animals. Biotechniques.

[20] Li W, Hansen L, Liu Y, Zemetra S, Berger H (2004). Using real-time PCR to determine transgene copy number in wheat. Plant Mol Biol Rep..

[21] Tong SK, Hsu HJ, Chung B (2010). Zebrafish monosex population reveals female dominance in sex determination and earliest events of gonad differentiation. Dev Biol..

[22] Siegfried R, Nüsslein-Volhard C (2008). Germ line control of female sex determination in zebrafish. Dev Biol..

[23] Nakamoto M, Matsuda M, Wang S, Nagahama Y, Shibata N (2006). Molecular cloning and analysis of gonadal expression of Foxl2 in the medaka *Oryzias latipes*. Biochem. Biophys. Res. Commun..

[24] Nishimura T, Tanaka M (2014). Gonadal development in fish. Sex Dev..

[25] Hu SY (2010). Nitroreductase-mediated gonadal dysgenesis for infertility control of genetically modified zebrafish. Mar Biotechnol..

[26] White YA, Woods DC, Wood AW (2011). A transgenic zebrafish model of targeted oocyte ablation and *de novo* oogenesis. Dev Dyn..

[27] Kurokawa H (2007). Germ cells are essential for sexual dimorphism in the medaka gonad. Proc Natl Acad Sci USA.

[28] Slanchev K, Stebler J, de la Cueva-Méndez G, Raz ES (2005). Development without germ cells: the role of the germ line in zebrafish sex differentiation. Proc Natl Acad Sci USA.

[29] Tzung KW (2015). Early depletion of primordial germ cells in zebrafish promotes testis formation. Stem cell Rep..

[30] Wong TT, Zohar Y (2015). Production of reproductively sterile fish by a non-transgenic gene silencing technology. Sci Rep..

[31] Dranow DB, Tucker RP, Draper BW (2013). Germ cells are required to maintain a stable sexual phenotype in adult zebrafish. Dev Biol..

[32] Köprunner M, Thisse C, Thisse B, Raz E (2001). A zebrafish nanos-related gene isessential for the development of primordial germ cells. Genes Dev.

[33] Gürcü B, Koca YB, Özkut M, Tuğlu Mİ (2016). Matrix changes due to the toxic effects of metronidazole in intestinal tissue of fish (*Onchorhynchus mykiss*). Chemosphere.

[34] Curado S (2007). Conditional targeted cell ablation in zebrafish: a new tool for regeneration studies. Dev Dyn..

[35] Hernández M, Gasca-Leyva E, Milstein A (2014). Polyculture of mixed-sex and male populations of Nile tilapia (*Oreochromis niloticus*) with the Mayan cichlid (Cichlasoma urophthalmus). Aquaculture.

[36] Liu H (2013). Genetic manipulation of sex ratio for the large-scale breeding of YY super-male and XY all-male yellow catfish (*Pelteobagrus fulvidraco* (Richardson)). Mar Biotechnol..

[37] Lee S, Katayama N, Yoshizaki G (2016). Generation of juvenile rainbow trout derived from cryopreserved whole ovaries by intraperitoneal transplantation of ovarian germ cells. *Biochem* &. Biophy Res Commun..

[38] Seki S (2017). Production of the medaka derived from vitrified whole testes by germ cell transplantation. Sci Rep..

[39] Ye H (2017). Establishment of intraperitoneal germ cell transplantation for critically endangered Chinese sturgeon *Acipenser sinensis*. Theriogenology.

[40] Silva MA (2016). Successful xenogeneic germ cell transplantation from Jundia catfish (*Rhamdia quelen*) into adult Nile tilapia (*Oreochromis niloticus*) testes. Gen Comp Endocr..

[41] Kawakami K (2004). A transposon-mediated gene trap approach identifies developmentally regulated genes in zebrafish. Dev Cell..

[42] Dong Z (2014). Improving the efficiency for generation of genome-edited zebrafish by labeling primordial germ cells. Int J Biochem Cell Biol..

[43] Hu P (2008). Retinoid regulation of the zebrafish *cyp26a1* promoter. Dev Dyn..

