# Supplementary material for: Generation of all-male-like sterile zebrafish by eliminating primordial germ cells at early development
Source: Sci Rep. 2018 Jan 30;8:1834. doi: 10.1038/s41598-018-20039-3 (PMC5789895; doi:10.1038/s41598-018-20039-3)
Supplement: Supplementary file 1 — Supplementary information [file 41598_2018_20039_MOESM1_ESM.doc]

Supplementary information

Generation of all-male-like sterile zebrafish by eliminating primordial germ cells at early development

Li Zhou^1^*, Yongyong Feng^1^*, Fang Wang^1^, Xiaohua Dong^2^，Lan Jiang^1^, Chun Liu^1^, Qinshun Zhao^2^, Kaibin Li^1^

^1^ Pearl River Fishery Research Institute, Chinese Academy of Fishery Sciences, No.1 Xingyu Road, Xilang, Liwan District, Guangzhou, Guangdong 510380, China.

^2^ MOE Key Laboratory of Model Animal for Disease Study, Model Animal Research

Center, Nanjing University, 12 Xuefu Road, Pukou High-tech Development Zone, Nanjing, Jiangsu 210061, China.

1. The original full-length gel for Figure 1C:


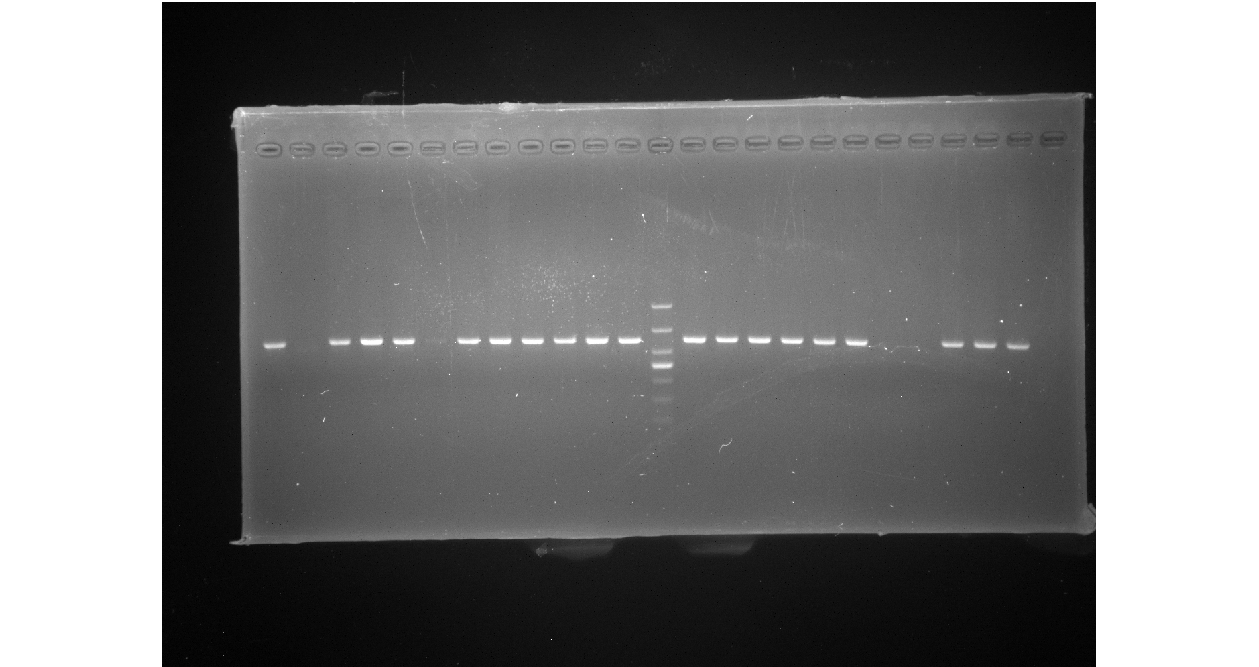


1. The original full-length gel for Figure 3G and 5N:


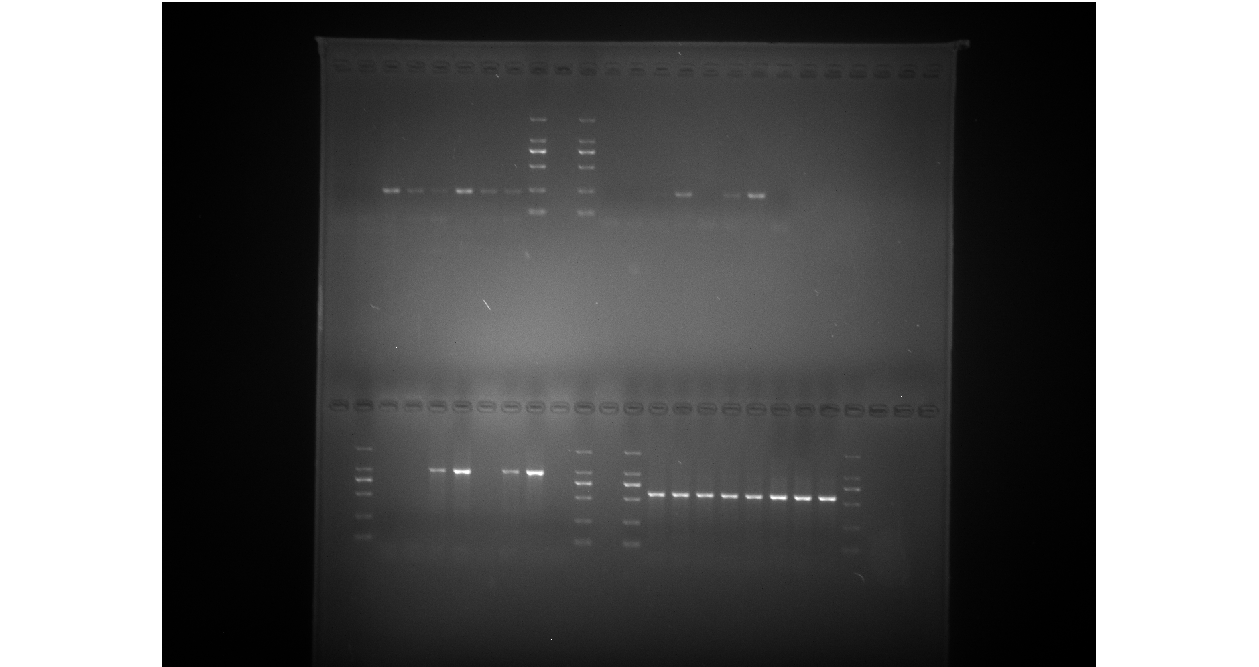


Gene *actb*: the gel image of Figure 3G was cropped from the red box, and Figure 5N was cropped from the green box.


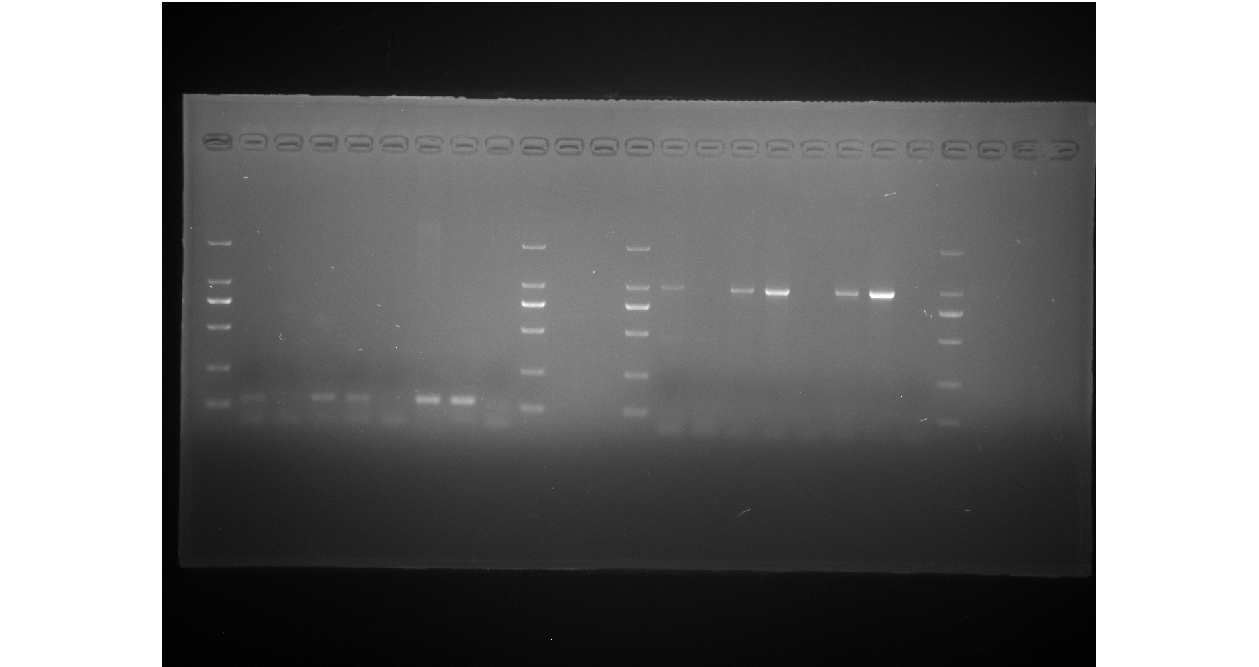


Gene *vasa*: Figure 3G in red box, Figure 5N in green box.

Gene *ziwi*: Figure 3G in blue box, Figure 5N in yellow box.


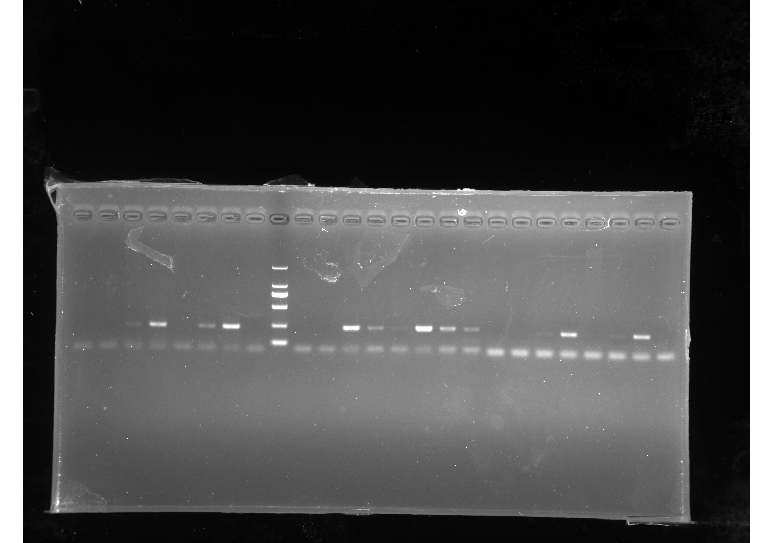


Gene *foxl2*: Figure 3G in red box, Figure 5N in green box.


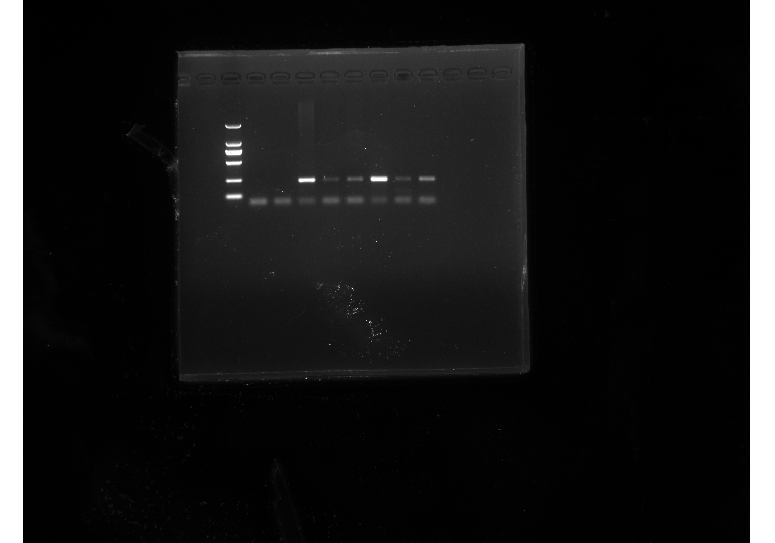


Gene *sox9a*: Figure 3G in red box, Figure 5N in green box.
